# The Efficacy and Safety of Anlotinib Combined With PD-1 Antibody for Third-Line or Further-Line Treatment of Patients With Advanced Non-Small-Cell Lung Cancer

**DOI:** 10.3389/fonc.2020.619010

**Published:** 2021-02-17

**Authors:** Chongya Zhai, Xiaoling Zhang, Lulu Ren, Liangkun You, Qin Pan, Hongming Pan, Weidong Han

**Affiliations:** Department of Medical Oncology, Sir Run Run Shaw Hospital, College of Medicine, Zhejiang University, Hangzhou, China

**Keywords:** NSCLC, anlotinib hydrochloride, third line of therapy, TP53, EGFR

## Abstract

**Background:**

Both anlotinib and programmed death 1 (PD-1) monoclonal antibody (mAb) have been approved for the third line treatment of metastatic non-small cell lung cancer (NSCLC). However, the combination of these two standard therapies has not been investigated in third-line or further-line treatment of patients with advanced NSCLC.

**Methods:**

We reviewed 22 patients with NSCLC who received anlotinib combined with PD-1 mAb therapy from July 2018 to October 2019 at Sir Run Run Shaw Hospital. Based on the baseline characteristics, PD-L1 expression and EGFR mutation status, we retrospectively analyzed the efficacy and safety of this combination therapy by RESIST 1.1 and CTCAE 5.0.

**Results:**

The combination treatment of anlotinib and PD-1 mAb in 22 NSCLC patients gained a median PFS of 6.8 months and a median OS of 17.3 months. The disease control rate (DCR) was 90.9%, and the objective response rate (ORR) was 36.4%, where 1 (4.6%) patient achieved complete response (CR) and 7 (31.8%) patients achieved partial response (PR). The median time to response was 3.9 months, and the median duration of the response was 6.8 months. The common grades 1–2 adverse events were fatigue 10/22 (45.5%), decreased appetite 9/22 (40.9%), hypertension 10/22 (45.5%); the common grades 3–4 adverse events were hypertension 2/22 (9.1%) and mouth ulceration 2/22 (9.1%).

**Conclusion:**

Anlotinib combined with PD-1 mAb showed promising efficacy in third-line or further-line treatment of NSCLC, and its adverse effects is tolerable.

## Introduction

Lung cancer is the leading cause of cancer-related morbidity and mortality, non-small-cell lung cancer (NSCLC) is the most prevalent subtype with a poor prognosis owing to the presence of locally advanced or wide metastasis in the majority of patients at the time of diagnosis or postoperative recurrence (1). Significant progress has been made in the treatment of advanced NSCLC in the past 10 years. In patients with positive drive mutation, the drugs represented by EGFR-TKI achieved nearly 3-year overall survival (2). For patients with negative drive mutation, PD-1 mAb can significantly improve the therapeutic efficacy and prolong the overall survival (3). In the first-line treatment, it can be used alone or combined with chemotherapy, while the second-line treatment is recommended to use PD-1 mAb alone (4). When PD-1 mAb is used alone, the overall response rate and PFS are not satisfactory (5–7). Some patients even suffered from hyper-progression with single immunotherapy due to high metastatic burden (8).

Anlotinib is a multi-target drug approved for the third line treatment of advanced NSCLC, which could inhibit the vascular endothelial growth factor (VEGFR) 1-3, platelet-derived growth factor (PDGFR) α, PDGFRβ, C-proto-oncogenic receptor tyrosine kinase (C-KIT) and RET. It represses the tumor angiogenesis by down-regulating major pro-angiogenic factors, such as VEGF, PDGF-BB and fibroblast growth factor (FGF). Surprisingly, anlotinib used in the third line treatment of NSCLC gained a median PFS of 5.4 months (9). Interestingly, one study has shown that anlotinib could enhance the ratio of CD8/FoxP3^+^ T cell in tumor tissues thus altering the tumor microenvironment (10). Moreover, anlotinib was proved to promote the infiltration of natural killer (NK) cells, M1-like tumor-associated macrophage (TAM) and dendritic cells in lung cancer mouse model, and the combination of anlotinib with immune checkpoint inhibitor gained better therapeutic response (11). These results suggest that anlotinib is involved in the regulation of tumor immune microenvironment, and the combination therapy with PD-1 mAb may be the future exploration direction.

There have been many reports on the treatment of NSCLC with PD-1 mAb combined with anti-angiogenetic agents. In patients with disease progression after first-line treatment, Sintilimab combined with bevacizumab showed unexpected efficiency. The combination therapy achieved a median PFS of 6 months, and the patients were well tolerated (12). Besides, Sintilimab combined with anlotinib in 22 patients of NSCLC at first line treatment gained an ORR of 68.2% and DCR of 100%, 2 grade 3 treatment-related adverse events (TRAEs) occurred with no grade 4/5 observation, however, the PFS and OS was not available due to the short follow-up time (13). Taken together, Angiogenesis inhibitors combined with immune check point inhibitors in the treatment of advanced lung cancer has achieved preliminary clinical validation.

Anti-angiogenesis agents and PD-1 mAb can cooperate to alter the microenvironment of the tumor. The expression of PD-1 was up-regulated in relapsed tumor after the anti-VEGFR2 agent treatment (14). Studies have shown that PD-1 mAb and anti-VEGFR2 agent combination treatment could promote tumor vessel normalization and induce high endothelial venules (HEVs), which promoted lymphocyte infiltration and activity through the activation of lymphotoxin β receptor (LTβR) signaling (15). In addition, anti-angiogenesis therapy could restore the response of effector T lymphocytes by breaking the tumor vessel barrier, and subsequent anti-PD-1 treatment further improved the activity of T lymphocytes, thereby synergistically leading to tumor shrinkage (16).

In this real-world evidence-based retrospective clinical study, we analyzed the efficacy and safety of anlotinib combined with PD-1 mAb for the third-line or further-line treatment of NSCLC patients. The results show that it has a good clinical application prospect and is worthy of further study.

## Methods

### Data Source

We reviewed the records of a prospectively collected database of 22 patients with NSCLC who received anlotinib combined with PD-1 mAb, including sintilimab, nivolumab, pembrolizumab, toripalimab, and camrelizumab for the third-line or further treatment over the period of 1 year from July 2018 to October 2019 at Sir Run Run Shaw Hospital, College of Medicine, Zhejiang University, Hangzhou, China.

We retrospectively analyzed the baseline characteristics, PD-L1 expression, epithelial growth factor (EGFR) mutation status and prior and later treatment lines. All data were obtained by follow-up visits, telephone, electronic medical records, and letters. This study was approved by the Ethics Committee of Sir Run Run Shaw Hospital.

### Patient Selection

**Figure 1:**
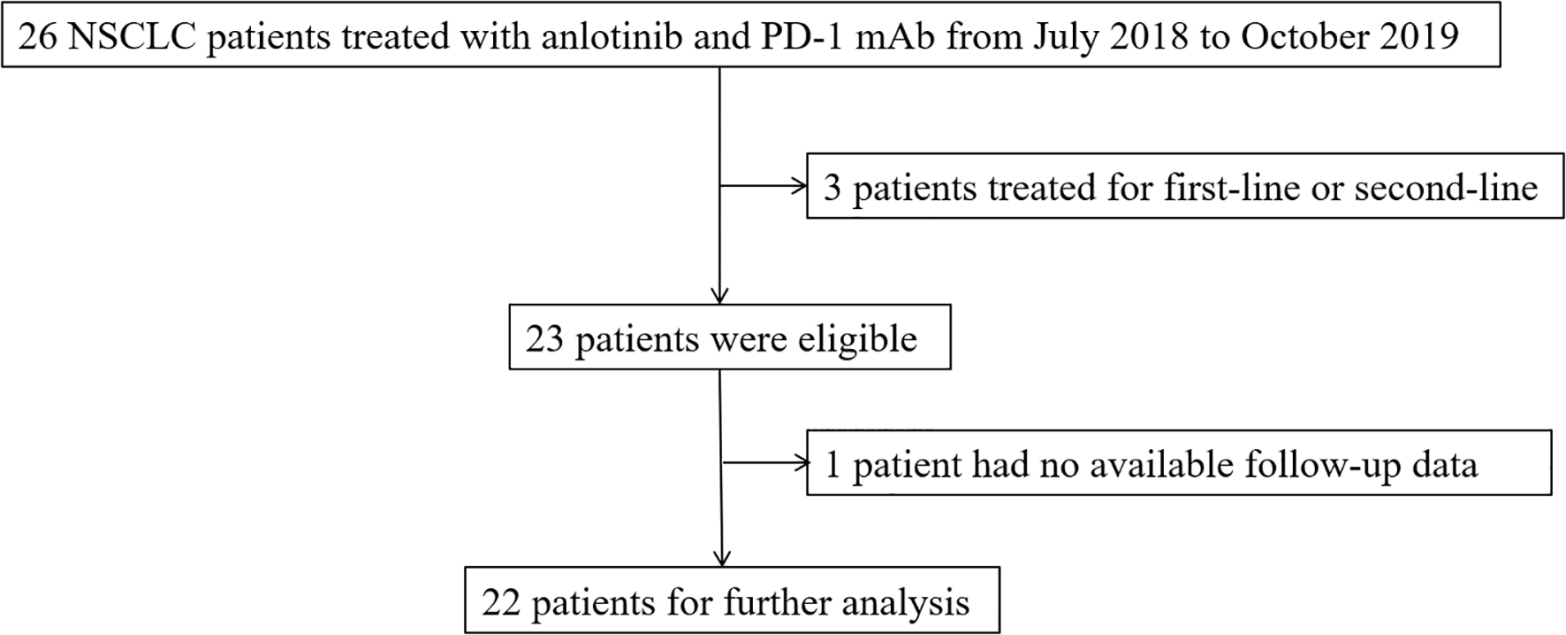
The patients selection flow chart.

The target samples included patients who received anlotinib and PD-1 mAb at Sir Run Run Shaw Hospital from July 2018 to October 2019. A definite histological or cytological diagnosis was required for the patients with NSCLC. The expected survival time was more than 3 months, and normal hematopoietic, hepatic, and renal function were prerequisite for enrollment. Exclusion criteria included patients with a history of autoimmune diseases or patients treated with steroids at a dose equivalent to or more than 10 mg prednisone daily or other immunosuppressive drugs. Patients with central squamous cell carcinoma were also excluded. The selection flow chart was shown in the **Figure 1**.

### Study Variables

The clinical response to anti-PD-1 mAb combined with anlotinib treatment was evaluated according to the Response Evaluation Criteria in Solid Tumors (RECIST) version 1.1. Every 2 or 3 cycles after the combined therapy was established, and short-term efficacy was evaluated. The percentage of patients having achieved a complete response (CR: the disappearance of all target lesions) plus partial response (PR: at least a 30% reduction in the sum of the diameters of the target lesions) recorded in the medical system were defined as the objective response rate (ORR). At least a 20% increase in the sum of the diameters of the target lesions were evaluated as PD, the lesions cannot be classified as PR or PD was evaluated as SD (stable disease). The percentage of patients with CR, PR or SD was defined as the disease control rate (DCR). PFS was calculated as the time from the initiation of treatment with anti-PD-1 mAb combined with anlotinib therapy to progressive disease (PD) or death. OS referred to the time from the start of combination treatment to death from any cause.

### Statistical Analysis

Descriptive statistics (percentages, means, medians) were used to describe baseline characteristics and clinical features of the sample of patients with NSCLC. ORR, PFS and OS were calculated to analyze the efficacy and clinical features of the patients treated with combination therapy. Statistical analyses were performed using SPSS statistical software (version 20.0; SPSS, IBM Corporation).

## Results

From July 2018 to October 2019, 22 patients with NSCLC were enrolled in this real-world study. Baseline demographics and clinical characteristics were exhibited in **Table 1**. In the final eligible sample, the median age of the patients was 65 years. Of 22, 10 (45.4%) patients were current or former smokers; 11 (50%), never smokers; and 1 (4.6%), unknown smoking status. Notably, 2 (9.1%) patients were with ECOG status of 0, and 20 (90.9%) patients had ECOG status of 1 at the time of diagnosis. 10 (45.4%) patients were current or former smokers, and 11 (50%) patients were never smoker. The smoking status was unknown in 1 (4.6%) patient. Patients with non-squamous histology predominated: 15 (68.2%) had adenocarcinoma, seven (31.8%) had squamous cell carcinoma. Fourteen (63.6%) patients had metastasis organ number more than 3 and eight (36.4%) patients had metastasis organ number below 3. As for the PD-1 mAb treatment, a numbers of patients received nivolumab, pembrolizumab, Sintilimab, toripalimab, or camrelizumab treatment were 6 (27.3%), 2 (9.1%), 5 (22.7%), 5 (22.7%) and 4 (18.2%), respectively. In addition, two (9.1%) patients had PD-L1 tumor proportion score (TPS) above 50%; seven (31.8%), below 1%; nine (40.9%), between 1% and 49%; four (18.2%), unknown PD-L1 TPS. The efficacy of PD-1 mAb was related to the basic EGFR mutation status. In this study, 12 (54.6%) patients had negative EGFR mutation status, followed by unknown EGFR mutation status (7, 31.8%), positive EGFR mutation (3, 13.6%), EGFR exon19 mutation (2, 9.1%), and EGFR L858/T790M mutation (1, 4.5%). 7 (31.8%) patients had radiotherapy previously and 3 (13.6%) patients ever received target therapy. The patients receiving 12 mg or 10 mg anlotinib dose numbered 6 (27.3%) and 16 (72.7%), respectively. The anlotinib dose was given after fully assessment of the tolerance and other basic physical status of the patients. The detailed anlotinib dose and treatment lines were listed in the **Supplementary Table 1**.

**Table 1:** Population characteristics.

Baseline Characteristics	All patients (n=22)
Age	
Median(range), years	65 (46–82)
Gender, n(%)	
Male	14 (63.6)
Female	8 (36.4)
ECOG score at the time of diagnosis, n(%)	
0	2 (9.1)
1	20 (90.9)
Histological subtype, n(%)	
Adenocarcinoma	15 (68.2)
Squamous cell carcinoma	7 (31.8)
Smoking status, n(%)	
Never smoked	11 (50.0)
Current or former smoker	10 (45.5)
Unknown	1 (4.5)
No.of prior systemic regimens, n(%)	
2	15 (68.2)
3	4 (18.2)
≥4	3 (13.6)
No.of organs of metastasis, n(%)	
≥3	14 (63.6)
<3	8 (36.4)
Anlotinib treatment dose, n(%)	
12mg	6 (27.3)
10mg	16 (72.7)
Anti-PD-1 mAbs, n(%)	
Nivolumab	6 (27.3)
Pembrolizumab	2 (9.1)
Sintilimab	5 (22.7)
Toripalimab	5 (22.7)
Camrelizumab	4 (18.2)
PD-L1 TPS, n(%)	
≥50%	2 (9.1)
1-49%	9 (40.9)
<1%	7 (31.8)
Unknown	4 (18.2)
EGFR mutation status, n(%)	
Negative	12 (54.6)
Positive	3 (13.6)
Unknown	7 (31.8)
Chemotherapy regimens, n(%)	
1	5 (22.7)
2	11 (50.0)
≥3	6 (27.3)
Previous radiotherapy treatment, n(%)	
No	15 (68.2)
Yes	7 (31.8)
Previous target treatment, n(%)	
No	19 (86.4)
Yes	3 (13.6)

Anti-PD-1, anti-programmed death-1; EGFR, epidermal growth factor receptor; ECOG, Eastern Cooperative Oncology Group; PD-L1, programmed death ligand-1; TPS, tumor proportion score.

**Figure 2:**
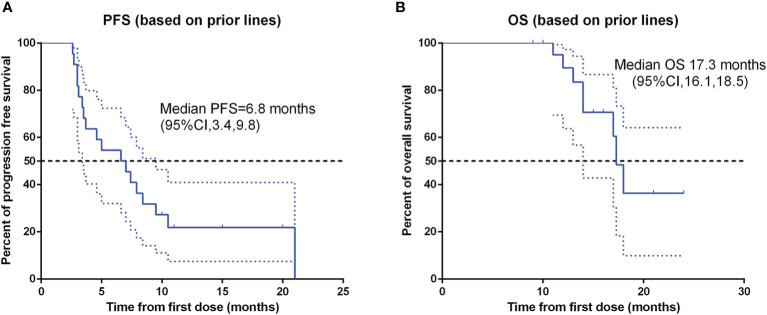
Kaplan-Meier survival curve of progression-free survival **(A)** and overall survival **(B)** in 22 non-small cell lung cancer patients. PFS, progression-free survival; OS, over survival; CI, confidence interval.

**Figure 3:**
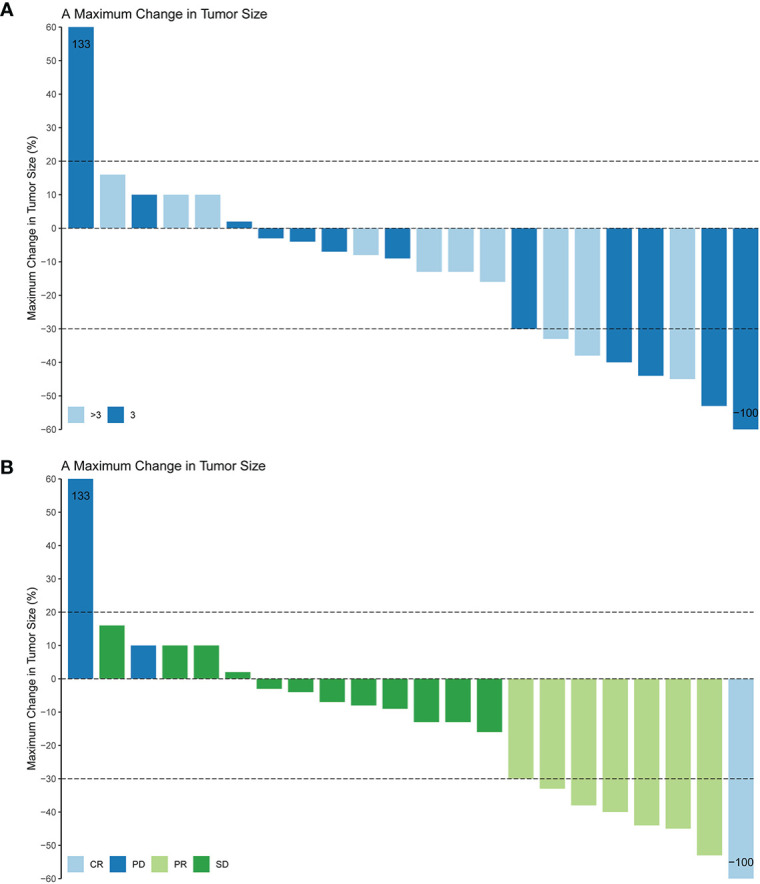
Maximum change in tumor size based on the treatment lines **(A)** and tumor response **(B)** in 22 non-small cell lung cancer patients. CR, complete response; PR, partial response; SD, stable disease; PD, Progressive disease; ORR, objective response rate.

**Figure 4:**
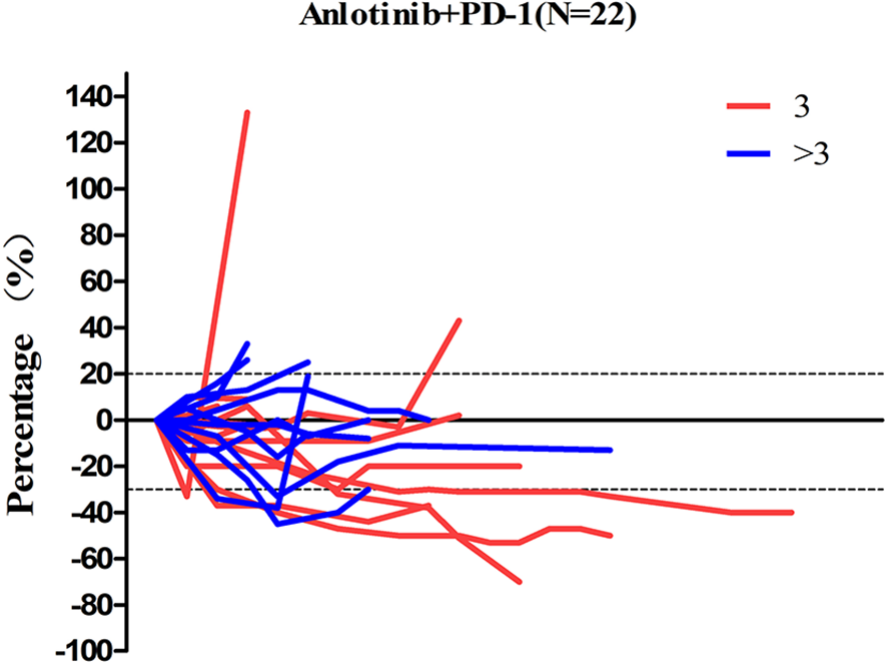
The percent change in the sum of the longest diameter of target lesions diameter from the baseline in 22 non-small cell lung cancer patients.

Patients response to anlotinib combined with PD-1 mAb was displayed in the **Table 2**. One (4.6%) patient got CR; seven (31.8%) patients got PR; 12 (54.5%) patients remained SD; and two (9.1%) patients had disease progressed. The ORR was 36.4% and the DCR was 90.9%. The median time to response was 3.9 months, and the median duration of the response was 6.8 months. The median PFS (**Figure 2A**) of the combination therapy was 6.8 months (95%CI: 3.4, 9.8), and the median OS (**Figure 2B**) of the treatment was 17.3 months (95%CI: 16.1, 18.5). For each patient, the percent change in the sum of the longest diameter of target lesions diameter from the baseline was graphed in a waterfall plot (based on the treatment lines and patient’s response, **Figures 3A, B**) and spider plot (**Figure 4**). For patients with brain metastasis subgroup analysis, six patients had brain metastasis, the median PFS was 4.7 months (95% CI:2.3–7.1 months), 16 patients had no brain metastasis, the median PFS was 10.5 months (95%CI:6.8–14.3 months), the *p* value is 0.053 between two groups using the log-rank survival analysis.

**Table 2:** Efficacy of Anlotinib combined with Anti-PD-1 mAbs in third-line or further treatment of NSCLC patients.

Efficacy	All patients (n=22)
Complete response, n(%)	1 (4.6)
Partial response, n(%)	7 (31.8)
Stable disease, n(%)	12 (54.5)
Progressive disease, n(%)	2 (9.1)
ORR (%, CR+PR)	36.4
DCR (%, CR+PR+SD)	90.9
Time to response(m)	
Media(range)	3.9 (1.6, 7.7)
Duration of response(m)	
Media	6.8
ongoing, n/N(%)	4/8 (50.0)
PFS(m)	
Media (95% CI)	6.8 (3.4, 9.8)
OS(m)	
Media (95% CI)	17.3 (16.1, 18.5)

CR, complete response; PR, partial response; SD, stable disease; PD, Progressive disease; ORR, objective response rate; DCR, disease control rate.

The adverse events during the combination treatment were listed in the **Table 3**. The most common grade 1-2 TRAEs were fatigue 10/22 (45.5%), decreased appetite 9/22 (40.9%), and hypertension 10/22 (45.5%). The less common mild adverse events were nausea 3/22 (13.6%), cough 2/22 (9.1%) and hepatic function abnormal 3/22 (13.6%). The grades 3–4 adverse events were rash 2/22 (9.1%), hypertension 2/22 (9.1%), diarrhea 1/22 (4.6%), mouth ulceration 2/22 (9.1%), and pneumonitis 2/22 (4.6%).

**Table 3:** Treatment-related adverse events.

AEs	Grade 1-2, n(%)	Grade 3-4, n(%)
Fatigue	10 (45.5)	0
Decreased appetite	9 (40.9)	0
Nausea	3 (13.6)	0
Weight decrease	4 (18.2)	0
Rash	8 (36.4)	1 (4.6)
Hypertension	10 (45.5)	2 (9.1)
Diarrhea	7 (31.8)	1 (4.6)
Mouth ulceration	5 (22.7)	2 (9.1)
Dysphonia	4 (18.2)	0
Pneumonitis	4 (18.2)	1 (4.6)
Cough	2 (9.1)	0
Hepatic function abnormal	3 (13.6)	0
Hypothyroidism	4 (18.2)	0
proteinuria	5 (22.7)	0
Palmar-plantar erythrodysaesthesia syndrome	4 (18.2)	0

AEs, adverse events.

## Discussion

From the retrospective summary of the anlotinib combined with the PD-1 mAb treatment, the efficacy was promising and unexpected. The third-line treatment of sole anlotinib in NSCLC gained a median PFS of 5.4 months and the median OS of 9.6 months (9), whereas combination treatment exhibited a median PFS of 6.8 months, median OS of 17.3 months and the ORR of 36.4%, providing an extra 1.4 months PFS and 7.7 months OS benefit compared with anlotinib alone. In comparison with sole PD-1 mAb treatment, the combination treatment also provided survival benefit. The median OS of nivolumab ranged from 9.2 months to 12.2 months in CheckMate 017 and CheckMate 057 clinical trials; 12.7 months of pembrolizumab in Keynote 010 clinical trial; for atezolizumab, the median OS ranged from 12.6 months to 13.8 months in OAK (third line therapy) and POPLAR clinical trial (6, 17). Therefore, the combination treatment gained an encouraging OS. This favorable OS was achieved for several reasons. The different treatment lines between Checkmate017, Checkmate057 (second-line) and our cohort (third line) were part of the reason for the prolonged OS, meanwhile, in standard third line treatment, anlotinib alone gained a favorable survival time, PD-1 mAb also prolonged the survival time compared with chemotherapy. Besides, the adverse events were tolerable for patients with combination treatment, few patients withdrew the clinical trial due to the TRAEs. With no doubt, the combination of PD-1 mAb and anlotinib can gained a longer survival benefit *via* a synergistic way. The mechanism may be that anlotinib altered the tumor immune microenvironment and PD-1 mAb improved the vessel normalization, which leads to the mutual sensitization between these two drugs (15). For instance, anlotinib treatment increased the INF-γexpression in CD4^+^ T cells and upregulated the tumor-infiltrating NK cells (11). Besides, anlotinib can inhibit PD-L1 expression on vascular endothelial cells so as to break through “immune tolerance barrier”, it also promotes CD8^+^ T cell infiltration and improves the balance of CD8/Foxp3 (10). Many pro-angiogenic factors are derived from immune cells, such as M2-like TAMs, immature dendritic cells, myeloid-derived suppressor cells (MDSCs), Trges and so on. These cells play various roles in the regulation of tumor angiogenesis (18). PD-1 mAb can suppress the activity of the immunosuppressive cells, indirectly down-regulate the angiogenic factors, and alleviate the abnormalities of tumor vessel (19, 20). In line with this, a number of experimental studies have confirmed that anti-angiogenesis agents combined with PD-1 mAb can reduce the tumor volume of tumor bearing mice (21, 22).

The median time to response (TTR) and the duration of response (DOR) of pembrolizumab in KEYNOTE 001 was 2.1 months and 18 months, respectively, and 101/495 (20.4%) patients in this clinical trial were treatment naïve (23). In camrelizumab (PD-1 mAb) combined with apatinib treatment, the TTR was 3.7 months and DOR was 5.3 months (24). While in our study, the TTR was 3.9 months, which is longer than the results of both above studies, probably because that most patients in this study were third line or above line and 63.6% (14/22) patients were with three or more organ metastases. Although the TTR was longer in our study, however, the DOR of our study is 6.8 months, which was lengthened than the camrelizumab combined with apatinib treatment. The results indicated that anlotinib combined with PD-1 mAb could also achieve long-term benefits.

It is worth noting that one patient achieved a CR response and had PFS for more than 20 months. The patient had complex mixed mutations, including K-RAS exon 2 missense mutation, TP53 exon 9 frameshift mutation, TAPBPL (TAP binding protein like) exon 2 missense mutation and SMARCA4 (SEI/SNF related, matrix associated, actin dependent regulator of chromatin, subfamily a, member 4) exon 20 missense mutation. Consistent with our findings, In Keynote 001, the median PFS of patients with K-RAS mutation and TP53 mutation treated with pembrolizumab was 14.7 months, much higher than that of 3.5 months in the wild-type group of K-RAS (25). Interestingly, it has been reported that a squamous-cell NSCLC patient with TP53 and KRAS co-mutation was treated with pembrolizumab combined with gemcitabine. The therapeutic effect reached PR and PFS was more than 7 months (26). The K-RAS mutation status may be an indicator of good response to the PD-1 mAb, the TP53 mutation patients also showed good response to anlotinib (27). So clinical trial like combination of anlotinib and PD-1 mAb may be promising in patients with K-RAS mutation and TP53 mutation co-existed. In addition, recently, it is generally believed that PD-L1 high expression is correlated with good prognosis in patients treated with PD-1 mAb (28, 29). In keynote 024, the patients whose PD-L1 TPS score >50% treated with pembrolizumab gained a median OS of 30 months (30), and in keynote-042 (TPS>50% Subgroup),the median OS is 20 months (31). It is impressive that in keynote 024, three patients treated with single pembrolizumab achieved CR (30, 32). In our study the CR patient also had a PD-L1 TPS above 50%. Therefore, among the three factors of K-RAS mutation, TP53 mutation and high score of PD-L1 TPS, which was the real prognostic factor is still uncertain. More clinical trials or experience should be focused on the factors.

For patients with brain metastasis, the prognosis was worse than the patients without brain metastasis, but the only 22 patients was included in our cohort, it may not be convincing enough until more prospective studies were conducted.

The adverse events of the combination treatment were tolerable in our patients, so the combination was safe and could be popularized in the future. The most common grade 1-2 adverse events were hypertension and fatigue (33), which were in line with the ALTER-0303 clinical trial. However, the thyroid stimulating hormone (TSH) elevation and hypertriglyceridemia were less common in our center, which might due to a limited number of cases. The common adverse events of PD-1 mAb were diarrhea, rash, decreased appetite, nausea, anemia and neutropenia (34). The organ specific immune-related adverse events were hypothyroidism, pneumonitis, colitis, hepatitis and hypophysitis (35). In our study, four (18.2%) patients had mild hypothyroidism, and four (18.2%) patients had grades 1–2 pneumonitis. These adverse events were consistent with the previous clinical trial. However, it should be noted that the median time to follow up was 384 days (95%CI: 298 days, 469 days), some immune related adverse event can occur later, for example, the hepatitis can occur after 34 weeks exposed to nivolumab treatment (36). The median time to onset of late-immune-related adverse events was 16.6 months in a multi-center study (37). So the immune-related adverse events need to follow up later.

Our study has obvious limitations because only 22 patients were included, resulting in the inability to carry out univariate or multivariate analysis. The anlotinib dose, different pathology types, number of metastasis organs, treatment lines and other factors may affect the efficacy of combination treatment. More clinical trials or clinical experience are needed to identify the beneficial patient group. In addition, retrospective study may lose some detailed information of the patients, like the gene mutation and PD-L1 of some patients were unknown; besides, multi PD-1 mAb were used in our center, which might produce diverse efficacy. In this study, three patients with EGFR mutation had a history of target therapy, the PFS of three patients were very different (3, 5, and 10.5 months, respectively). So we cannot come to a conclusion right now, this requires more data based on these subtype of patients. To sum up, our retrospective analysis shows that the efficacy and safety of the combination therapy of anlotinib and PD-1 mAb are encouraging and worthy of further clinical trials.

## Data Availability Statement

The raw data supporting the conclusions of this article will be made available by the authors, without undue reservation.

## Ethics Statement

Written informed consent was obtained from the individual(s), and minor(s)’ legal guardian/next of kin, for the publication of any potentially identifiable images or data included in this article.

## Author Contributions

CZ: Data curation, Methodology, Investigation. XZ: Data curation, Methodology, Writing original draft. LR: Data curation, Writing-review and editing. LY: Data curation, Methodology. QP: Methodology, Supervision. HP: Methodology, Supervision. WH: Methodology, Supervision, Writing-review and editing. All authors contributed to the article and approved the submitted version.

## Funding

This work was supported by the National Natural Science Foundation of China (81972745 and 81703072), the Ten Thousand Plan Youth Talent Support Program of Zhejiang Province (ZJWR0108009), and the Zhejiang Medical Innovative Discipline Construction Project-2016, and the Hangzhou Health and Family Planning and Science and Technology Program (OO20190347).

## Conflict of Interest

The authors declare that the research was conducted in the absence of any commercial or financial relationships that could be construed as a potential conflict of interest.
