# Supplementary material for: The Efficacy and Safety of Anlotinib Combined With PD-1 Antibody for Third-Line or Further-Line Treatment of Patients With Advanced Non-Small-Cell Lung Cancer
Source: Front Oncol. 2021 Feb 17;10:619010. doi: 10.3389/fonc.2020.619010 (PMC7927598; doi:10.3389/fonc.2020.619010)
Supplement: Supplementary file 1 [file DataSheet_1.doc]

| Patient No. | sex | age (years) | Histological subtype | ECOG | EGFR mutation status | PD-L1 status | No.of organs of metastasis | TNM stage | Treatment initiation time | Treatment line | Anlotinib dose(mg) | Anti-PD-1 mAbs | Anti-PD-1 mAbs dose(mg) | Efficacy | PFS(m) | OS(m) |
| --- | --- | --- | --- | --- | --- | --- | --- | --- | --- | --- | --- | --- | --- | --- | --- | --- |
| 1 | female | 65 | adenocarcinoma | 1 | EGFR19 | 10%-50% | 4 | cT1N0M1c | 2018/7/20 | 8 | 10 | camrelizumab | 200 | SD | 10.5 | 17.3 |
| 2 | male | 70 | squamous carcinoma | 0 | unknown | negative | 3 | cT1N0M1c | 2018/8/29 | 3 | 10 | nivolumab | 200 | PR | 21 | alive |
| 3 | male | 82 | adenocarcinoma | 1 | K-RAS TP53 | ＞50% | 1 | cT3N0M1a | 2018/11/26 | 3 | 12 | toripalimab | 240 | CR | ＞20 | alive |
| 4 | male | 60 | adenocarcinoma | 1 | none | negative | 1 | cT4N2M1a | 2019/4/25 | 4 | 12 | sintilimab | 200 | PR | 7.9 | alive |
| 5 | female | 55 | adenocarcinoma | 1 | EGFR19 | negative | 3 | cT4N3M1c | 2019/2/28 | 3 | 10 | sintilimab | 200 | SD | 3 | 3.4 |
| 6 | male | 68 | adenocarcinoma | 1 | none | 1-10% | 4 | cT2N2M1c | 2019/3/5 | 3 | 12 | pembrolizumab | 200 | PD | 2.6 | 2.6 |
| 7 | female | 67 | adenocarcinoma | 1 | ROS1 | negative | 3 | cT2bN3M1c | 2019/2/26 | 3 | 12 | sintilimab | 200 | SD | 9.5 | alive |
| 8 | male | 62 | squamous carcinoma | 1 | unknown | Unknown | 1 | cT4N2M1a | 2019/3/14 | 4 | 12 | nivolumab | 200 | SD | 3.1 | alive |
| 9 | female | 46 | adenocarcinoma | 1 | none | 1-10% | 1 | cT4N2M1a | 2019/4/27 | 4 | 10 | sintilimab | 200 | PR | 7.4 | alive |
| 10 | male | 70 | squamous carcinoma | 1 | unknown | 1-10% | 1 | cT4N3M1b | 2019/4/1 | 3 | 10 | nivolumab | 200 | PR | ＞15 | alive |
| 11 | female | 71 | adenocarcinoma | 1 | none | Unknown | 4 | cT2N2M1c | 2019/11/6 | 6 | 10 | toripalimab | 240 | SD | 7 | alive |
| 12 | male | 55 | adenocarcinoma | 1 | none | Unknown | 5 | cT4N2M1c | 2019/6/12 | 3 | 10 | nivolumab | 200 | SD | 3.7 | 5.4 |
| 13 | female | 57 | adenocarcinoma | 1 | none | negative | 3 | cT4N2M1c | 2019/6/27 | 3 | 10 | nivolumab | 200 | PR | 4.6 | 5.8 |
| 14 | male | 57 | adenocarcinoma | 1 | none | 10%-50% | 2 | cT4N2M1c | 2019/8/8 | 3 | 12 | nivolumab | 200 | PR | ＞11 | alive |
| 15 | male | 65 | adenocarcinoma | 1 | none | negative | 5 | cT2N2M1c | 2019/7/12 | 3 | 10 | toripalimab | 240 | SD | 3.5 | 8 |
| 16 | male | 75 | squamous carcinoma | 1 | unknown | 5% | 5 | cT2NxM1c | 2019/8/2 | 3 | 10 | toripalimab | 240 | PD | 2.7 | 5 |
| 17 | male | 61 | squamous carcinoma | 0 | unknown | 10%-50% | 1 | cT3N2M1a | 2019/9/24 | 3 | 10 | toripalimab | 240 | PR | ＞10 | alive |
| 18 | female | 63 | adenocarcinoma | 1 | EGFR L858R T790M | negative | 4 | cT1N2M1c | 2019/7/10 | 5 | 10 | pembrolizumab | 200 | SD | 5 | alive |
| 19 | male | 68 | adenocarcinoma | 1 | none | Unknown | 3 | cT3N2M1c | 2019/5/24 | 4 | 10 | sintilimab | 200 | SD | 3.4 | alive |
| 20 | female | 77 | adenocarcinoma | 1 | none | 5% | 3 | cT2N2M1c | 2019/10/11 | 3 | 10 | camrelizumab | 200 | SD | 6.6 | alive |
| 21 | male | 69 | squamous carcinoma | 1 | unknown | 1-10% | 3 | cT4N2M1c | 2019/9/27 | 3 | 10 | camrelizumab | 200 | SD | 3 | 5.8 |
| 22 | male | 65 | squamous carcinoma | 1 | unknown | ＞50% | 2 | cTxN3M1c | 2019/9/29 | 3 | 10 | camrelizumab | 200 | SD | 8.4 | alive |

These statistics are updated to July 2020.
